# Assessing clinical and morphological features of megalotrichosis induced by Tyrosine kinase inhibitors versus Prostaglandins analogues

**DOI:** 10.1371/journal.pone.0313326

**Published:** 2024-12-31

**Authors:** Rotem Gershon, Vicktoria Vishnevskia-Dai

**Affiliations:** 1 Faculty of Medicine, Tel-Aviv University, Tel-Aviv, Israel; 2 The Goldschleger Eye Institute, Ocular Oncology Service, Sheba Medical Center, Ramat-Gan, Israel; City University of New York, UNITED STATES OF AMERICA

## Abstract

**Purpose:**

Describing the features of Megalotrichosis (MT) induced by Tyrosine kinase inhibitors (TKI) and differentiate it from Prostaglandins (PGs)–induced MT.

**Methods:**

Medical data of patients with MT referred to our center between 2012–2021 were retrieved for: demographic parameters, medical, surgical and oncologic background, and ophthalmologic background along with diagnoses and treatment. Time from PGs/TKI introduction to MT presentation, MT clinical characteristics, associated complaints, and prescribed therapies in relevant cases were also documented. Ophthalmologic exam, ocular photography and data retrieved from medical records were used to assess MT features among the two groups. Morphological evaluation included number of upper (UL) and lower lid (LL) eyelash rows, poliosis, individual elongated eyelash and eyelash curvature. Masked evaluation of all the patients was performed.

**Results:**

We found 11 patients, of which 6 treated with PGs for glaucoma and 5 treated with TKIs for non-ocular cancer suspected of dissemination. TKIs-induced MT was characterized by more individual elongated eyelashes (p = .047), UL eyelash rows (p = .03) and eyelash curvature (p = .076); poliosis characterized PGs-induced MT (p = .076). MT-associated complaints were more frequent in TKIs-induced MT (p = .06). time from drug administration to MT onset was shorter with TKI compared to PGs (median 176 Vs. 440 days, p = .257).

**Conclusions:**

The study suggests that TKI-induced MT presents faster than PGs-induced MT and might be more bothering to patients. Knowledge of the morphological and clinical features that characterize each form of MT might be beneficial for patients and guide clinicians for intervention when needed. Larger cohorts are needed to reproduce and clarify our findings.

## Introduction

Eyelash megalotrichosis (MT) is an overgrowth of eyelashes [1]. This rare phenomenon can be a manifestation of congenital syndromes and diseases, or acquired condition secondary to malignancy, infection (i.e., HIV), and autoimmune diseases, or side effect of therapy with several pharmaceutical agents [2,3]. MT was reported as a side effect of treatment with Prostaglandin analogues (PGs) and Tyrosine Kinase Inhibitors (TKIs) [1]. PGs are a group of agents used as topical ophthalmologic treatments (drops) for glaucoma that reduce intra-ocular pressure (IOP) [4]. PGs-induced MT is a common and well-documented side effect. The PGF2α-analogue bimatoprost increases the number of eyelashes and induces hair shafts thickening and elongation in murine models [5]. In human, 26%-76% of glaucoma patients treated with the PGs latanoprost, bimatoprost or travoprost experience eyelash growth, per different agents [4]. TKIs are small molecules which inhibit growth factor receptors pathways, epidermal mainly, and are widely used in several cancers therapy with non-small cell lung cancer (NSCLC) being the most prominent [6]. These agents have several dermatologic and ophthalmologic side effects, which among others include MT [6]. TKIs-induced MT was reported among patients treated with erlotinib [7,8] and gefitinib [9]. it was also reported following administration of monoclonal antibodies targeting epidermal growth factor receptors (EGFR) as cetuximab and panitumumab [10,11]. A retrospective study on ocular toxicities among 69 patients treated with EGFR inhibition for malignancy found that 22 patients developed MT or trichiasis in 12 months, of which 9 (13%) required removal or trimming of eyelashes due to irritation and 2 (2.9%) suffered corneal abrasion [12]. Of 42 patients treated with the TKI erlotinib, 12 (28.6%) developed MT or trichiasis (growth of lashes towards the eye) [12]. Thus, MT might be a disturbing side effect not only cosmetically, but also due to risk for corneal irritation, abrasion, and the need for consequent ophthalmologic intervention [12]. TKIs-induced MT was mainly described in discrete case reports [7–11]; As both PGs-treated glaucoma and TKIs-treated cancers are common health conditions [4,12], substantial portion of patients will develop MT as an adverse effect. Our study aims to improve clinical and morphological recognition of different forms of MT, and thus improve awareness and clinical care of patients with this condition.

## Methods

### Patients

The study was approved by the Sheba Tel-Ha’Shomer Medical Center ethics committee (Helsinki committee). No consent was requested due to the retrospective nature of the study. This retrospective observational case series included 11 patients who were referred to the ocular oncology Service, The Goldschleger eye institute, Sheba medical center, between the years 2012–2021 for evaluation of suspected ocular oncological conditions and were found to suffer of megalotrichosis (MT). Clinical data were retrieved from the hospital medical registry. Data were accessed on February 25^th^ 2023, the authors had no access to any identifiable information during data collection.

### Medical evaluation

Patients’ age, gender, ethnicity, medical background, surgical background, oncologic background and ophthalmologic background along with past and present medical therapies were retrieved from hospital’s medical registry. time from PGs/TKIs introduction to MT presentation was also documented, as well as MT clinical characteristics, associated complaints, and prescribed therapies in relevant cases.

### Ophthalmologic evaluation

All patients underwent full ophthalmologic evaluation, which included best corrected visual acuity, intraocular pressure measurement, slit lamp examination of the anterior segment and posterior sement examination with dilated pupils. Best corrected visual acuity was measured with a standard Snellen chart and intraocular pressure was measured with a standard Goldman tonometer. Visual acuity parameters are reported at the logMAR scale.

### Ocular photography

All patients except one, from the TKIs group who had detailed description in the medical records, were photographed using Slit lamp camera (Topcon CORE BGI UNT BG-4). Clinical characteristics of MT were evaluated independently by VD and RG in a masked fashion without awareness of the treatment background (PGs or TKIs). Morphological features to characterize MT included number of upper lid eyelash rows (UL rows), number of lower lid eyelash rows (LL rows), poliosis (depigmentation of eyelashes), trichiasis (misdirection of eyelashes toward the globe), individual elongated eyelashes (defined as ≥2 times the length of regular eyelash), and eyelash curvature (defined as >1 growth angle change along a single eyelash).

### Statistical analysis

All statistical analyses were made using R software for statistical programming, version 3.6.3. tables production was made using the Tableone package. Categorical variables are described as N (%), and continuous variables are described as median [range]. Due to low number of samples, we used Fisher’s exact test to assess dependence between two categorical variables, and Mann-Whitney-Wilcoxon test to assess difference between two continuous variables. For all hypotheses testing, we declared p < .05 as significant result.

## Results

### Patients’ characteristics

Of 11 patients, 6 had prior glaucoma treated with PGs, and were referred to the clinic due to suspected or diagnosed ophthalmological malignancy. Their median age was 71 [range: 60–76], 3 were females and 3 were males; 3 patients were referred due to ocular lesions suspected of malignancy, 2 had prior diagnosis of choroidal melanoma, and 1 had prior skin melanoma which was suspected of ocular dissemination.

5 patients diagnosed with primary non-ophthalmologic metastatic malignancy treated with TKIs were referred to the clinic for ophthalmologic evaluation. their median age was 60 [range: 38–71], 3 were females and 2 were males; 4 patients had no prior ophthalmologic disease, and 1 was priorly diagnosed and treated with PGs for open angle glaucoma and is thus excluded from some analyses. Among PGs group, one patient had gout, and another one had Sjogren’s disease. Among the TKIs group, one patient had ischemic heart disease requiring percutaneous catheterization and stenting.

### Ophthalmologic evaluation

All Patients were evaluated by a specialist in ocular oncology from the Ocular Oncology service, The Goldschleger eye institute, Sheba medical center. Visual acuity (VA) and Intra-Ocular Pressure (IOP) did not show any significant differences between the two groups; pupil and iris examination were normal for all patients. Since the PGs glaucoma patients were referred due to suspected or diagnosed malignant ocular lesions, major differences exist between the two groups regarding fundus examination (not shown). Higher proportion of TKIs-treated patients presented with MT-associated complaints (3/5 Vs. 0/6 patients in the PGs-group, p = .06). However, no significant difference was found in proportion of patients needed lubrication therapy (2/5 TKIs-treated Vs. 1/6 PG-treated, p = .55).

**Table 1** summarizes patient’s characteristics among PGs and TKIs groups and demonstrates that no significant differences were found between the groups regarding demographic, clinical or ophthalmologic exams, apart from MT-related complaints and need for therapy.

**Table 1:** Patients’ characteristics.

	PGs treatment	TKI treatment	P	Overall
N	6	5		11
**Age at diagnosis** (median [range])	70.95 [60.1,75.5]	60.4 [38.1,71.2]	0.126	68.7 [38.1, 75.5]
**Male gender** (%)	3 (50)	2 (40)	1	5 (45.5)
**Origin** (%)	2 (33.3)	3 (60)	0.782	
Ashkenazi	2	3		
Mediterranean	3	2		
Other	1			
**Malignancy**				
NSCLC		4		
TCC		1		
**Malignancy treatment**				
Osimertinib		2		
Other TKI ^a^		3		
**Glaucoma**				
Open-angle glaucoma	5			
Angle-closure glaucoma	1			
**Glaucoma treatment** ^b^				
Travoprost-containing	4			
Latanoprost/Bimatoprost	2			
**MT complaint** (%)	0 (0)	3 (60)	0.06	3 (27.3)
**MT treatment** (%)	1 (16.7)	2 (40)	0.55	3 (27.3)
**Ophthalmologic exam** (median [range])				
VA left (logMAR)	0.1 [0,0.8]	0 [0,0.7]	0.631	0.1 [0,0.8]
VA right (logMAR)	0.1 [0,0.2]	0 [0,0.2]	0.407	0 [0,0.2]
IOP left	16 [12,20]	16 [10,17]	0.58	16 [10,20]
IOP right	17 [12,18]	16 [10,19]	0.674	16 [10,19]

^a^ Other TKI included: Efatinib, Erlotinib, experimental FGFR inhibitor (1 patient received each).

^b^ All glaucoma patients except one had past or concurrent glaucoma therapy with other agents, including alpha agonists, beta antagonists, carbonic anhydrase inhibitors and cromolyn. Travoprost-containing regimens included travoprost-timolol (duotarv) and single-agent travoprost (travatan). 1 patient received latanoprost and 1 received bimatoprost. **PG**s–Prostaglandin analogues, **TKI**–Tyrosine Kinase Inhibitor, **NSCLC**–Non-Small Cell Lung Cancer, **TCC**–Transitional (urothelial) cell cancer, **MT**–Megalotrichosis, **VA**–Visual Acuity, **IOP**–Intra-Ocular Pressure.

### Time from therapy introduction to MT presentation

Time from drug introduction to MT presentation was shorter with TKIs (Median 176 [range: 56–418] days) compared to PGs (Median 440 [155–878] days). Wilcoxon rank-sum test did not find significant difference in time to MT presentation (p = .257) between the two groups.

### Morphological assessment of MT

**Fig 1:**
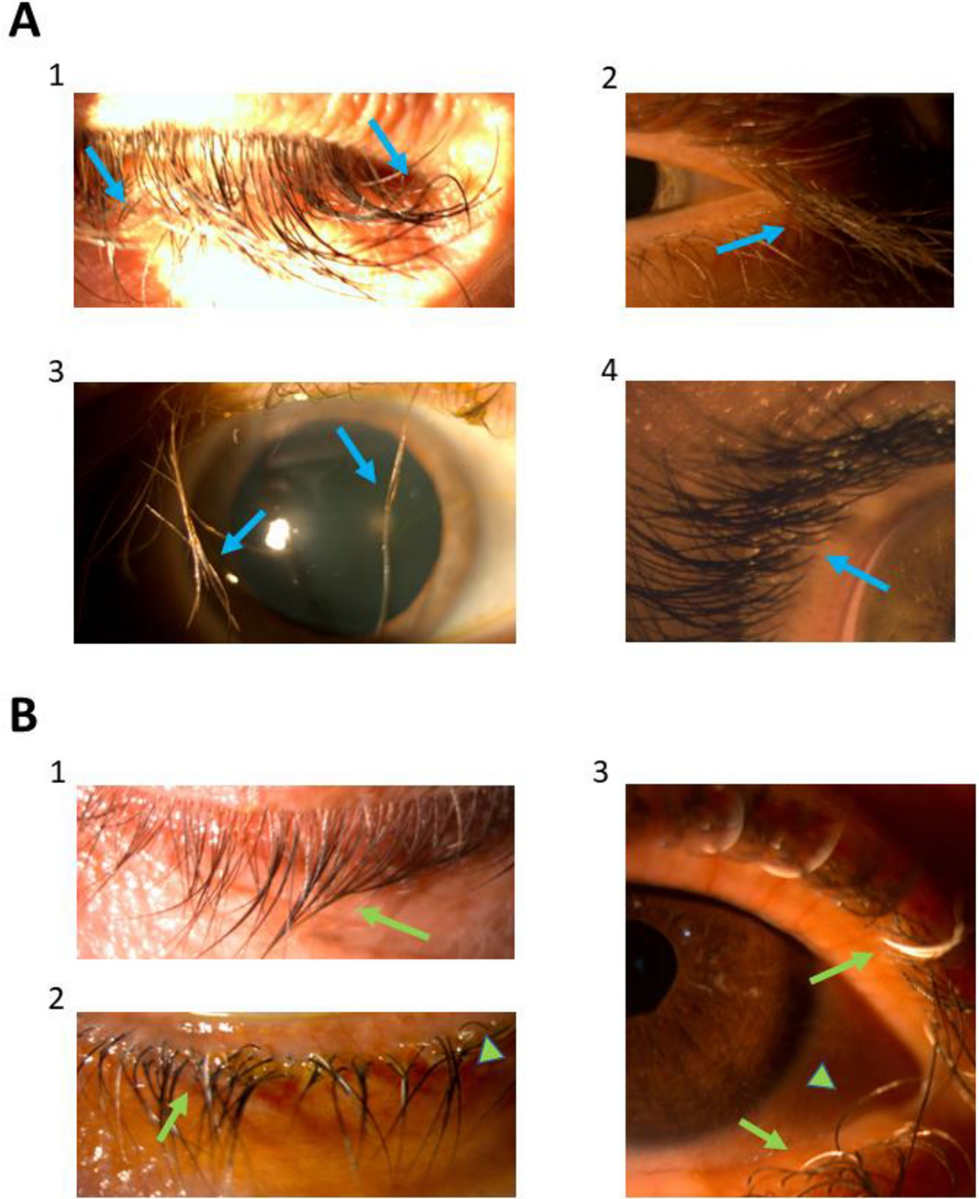
Morphological features of (A) TKIs-induced and (B) PGs-induced MT. **(A)** representative features of TKIs-induced MT (blue arrows): (1) elongated individual eyelashes with curvature (2) increased UL rows of elongated lashes (3) elongated, curved lashes with trichiasis (4) increased UL eyelashes rows; **(B)** representative features of PGs-induced MT (green arrows) (1) non-curved elongated lashes (2) non-curved lashes with poliosis (depigmented lashes, arrows) and trichiasis (arrowhead) (3) poliosis (arrows) and trichiasis (arrowheads). TKIs–Tyrosine Kinase Inhibitors; PGs–Prostaglandin analogues; MT–Megalotrichosis; UL–Upper Lid.

For each patient, assessment of MT morphological features was conducted independently by VD and RG. The features assessed on morphological examination of MT included poliosis, trichiasis, number of eyelash rows on LL and UL, individual elongated eyelash (defined as ≥2 times the length of regular eyelash) and eyelash curvature based on the detailed description in the medical record and/or slit lamp photographs. Representing pictures, with prominent morphological features are depicted in **Fig 1**. TKIs-treated cancer patients had significantly more individual elongated eyelashes (4/4 on TKIs group Vs. 1/6 on PGs group, Fisher’s exact p = .047) and number of UL eyelash rows (medians: 5.5 on TKIs group Vs. 4 on PGs group, Wilcoxon rank-sum p = .03). Trends toward significance were found regarding higher poliosis proportion among PGs-treated patients (0/4 on TKIs group Vs. 4/6 on PGs group, Fisher’s exact p = .076) and lower proportion of eyelash curvature among PGs-treated patients (4/4 on TKIs group Vs. 2/6 on PGs group, Fisher’s exact p = .076). Trichiasis and number of LL eyelash rows did not differ between the two groups. **Table 2** summarizes morphological features comparison between the two groups and time from drug introduction to MT presentation.

**Table 2:** Comparison of MT morphological features and drug-MT interval between patients with PGs-treated glaucoma and patients with TKI-treated malignancy.

	PG treatment	TKIs treatment	p	Overall
**N**	**6**	**4**		**10**
**Poliosis** (%)	4 (66.7)	0 (0.0)	0.076	4 (40.0)
**Elongated hairs** (%)	1 (16.7)	4 (100.0)	0.047	5 (50.0)
**Eyelash curvature** (%)	2 (33.3)	4 (100.0)	0.076	6 (60.0)
**Trichiasis** (%)	2 (33.3)	3 (75.0)	0.524	5 (50.0)
**UL rows** (median [range])	4 [4,5]	5.5 [5,6]	0.03	5 [4,6]
**LL rows** (median [range])	3.5 [3,5]	3.5 [3,4]	0.9	3.5 [3,5]
**Drug-MT interval** (median [range])	440 [155, 878]	176 [56,418]	0.25	209.5 [56,878]

**MT**–Megalotrichosis, **PG**–Prostaglandins, **TKIs**–Tyrosine Kinase Inhibitors, **UL**–upper eyelid, **LL**–lower eyelid.

## Discussion

Glaucoma treated with PGs and cancer treated with TKIs are common health conditions; Up to 76% of glaucoma patients treated with PGs [4] and 28.6% of patients with TKIs-treated malignancy [12] might develop MT as a side effect of their therapy. While MT is a common, well-documented, sometimes desired side effect of PGs treatment with minor clinical significance [13], clinical effects of TKIs-induced MT were not systematically evaluated and as this study suggests it might be of more clinical importance. In our center, 11 patients were referred for evaluation in the Ocular Oncology service of suspected conditions over a period of 10 years and were found to suffer of MT. The discrepancy between the high reported rates of MT among PGs or TKIs-treated patients and the low numbers of patients in our series might represent referral bias and possibly imply that most MT cases are asymptomatic and are diagnosed as accidental finding or have mild symptoms treated in primary care centers. Another potential limitation of the study is that glaucoma patients were referred to ocular oncology department due to background non-ocular malignancy suspected of dissemination; thus, they may not well represent the general population of patients with glaucoma.

Although reports on the prevalence and incidence of MT developing as side effect of PGs or TKIs treatment are scarce in the literature, this study focused on characterizing disparities between these two forms of MT, and due to above mentioned limitations, describing the epidemiological features of this phenomenon was not part of this study.

A previous study analyzed ocular toxicities among patients treated with EGFR inhibition who were referred to the cornea service, over a period of 5 years [12]. In total, 22 patients presented with MT or trichiasis; nonetheless, only 9/22 (40.9%) patients required intervention, a proportion similar to our 2/5 (40%) patients who received therapy for TKIs-induced MT. Similar data regarding PGs-induced MT complaints or need for treatment were not found by the authors, hence comparison was not available. However, in our cohort 3/5 patients treated with TKIs had MT-related complaints, as opposed to 0/6 patients treated with PGs. This finding may imply that TKIs-induced MT might be more disturbing to the patient than PGs-induced MT. Interventions reported in the literature for TKIs-induced MT include topical lubrication agents and eyelash trimming in extreme cases [12]. In our experience, 2 patients received topical preservative-free vitamin A-containing lubrication ointment, and 1 of these patients also needed eyelash trimming to alleviate corneal irritation due to MT.

Despite our small sample sizes, significant or near-significant differences distinguish each form of MT: TKIs-induced MT is characterized by more individual elongated eyelashes, higher number of UL eyelashes rows, and eyelash curvature; PGs-induced MT had higher proportion of poliosis. While to our knowledge reports on morphological features of TKIs-induced MT are absent in the literature, Johnstone described hypertrichosis among 43 patients treated with PGs (latanoprost) drops [14]; Concordant with our work, he noted increased eyelash rows and eyelash curvature as morphological features of latanoprost-induced MT. The study also noted increased eyelash pigmentation as a side effect; opposingly, and compatible with our findings, Chen et al. reported 7 cases of poliosis following topical Prostaglandin F2-α (PGF2α) administration for primary open-angle glaucoma [15].

In our study, time from drug administration to MT onset was 440 days [range:155–878] among PGs-treated group and 176 days [56–418] among TKIs-treated group. The difference was not found statistically significant, probably due to small cohort. Larger cohorts are needed to validate this trend. Studies documenting the time interval from drug to MT onset are scarce. Patients at Johnstone’s cohort were treated with latanoprost for at least 10 weeks (mean:19.8, range:11–40) before evaluating latanoprost-induced MT [14], yet time to onset is not specifically documented. A paper reviewing several clinical trials on PGs for treating glaucoma reported eyelash growth as adverse effect 3, 6, 9, and 12 months after starting PGs therapy [4], yet no case was reported among 1-month trials. Although not stated by authors, MT was more prevalent with longer duration of use [4]. Consistent with our results, A study describing ocular toxicities of EGFR inhibition in patients with malignancy noted that all cases of trichomegaly occurred within 3–5 months after starting therapy [12]. However, trichomegaly and trichiasis were reported simultaneously, and times to MT were not stratified to therapy as both TKIs and monoclonal antibodies were used to inhibit EGFR. Consequently, time interval from TKIs administration to MT onset was not reported.

Several other medical conditions (e.g. congenital syndromes, autoimmune diseases, HIV infection) and therapeutic agents (e.g. Calcineurin inhibitors, topiramate) are reported in the literature as potential inducers of MT [1,13]. However, PGs and TKIs induced MT have the highest quality support in literature while MT association with other medical conditions or therapies have lower quality of evidence [13]. In our cohort, over 10 years of follow-up, 11 cases of MT were diagnosed and treated following the use of PGs or TKIs. None of the patients had other medical conditions or therapies suspected to induce MT. Hence, despite potential clinical relevance, the features of MT induced by other medical conditions and medications are beyond the scope of this study.

## Supporting information

S1 Table Clinical and morphological features of MT patients. (XLSX)

## References

[1] Vij A, Bergfeld WF. Madarosis, milphosis, eyelash trichomegaly, and dermatochalasis. *Clin Dermatol*. 2015;33(2):217–226. doi: 10.1016/j.clindermatol.2014.10.013 25704941

[2] P M-P, EW C. Trichomegaly induced by topical tacrolimus for the treatment of periorbital vitiligo: A brief report of a new adverse effect. *Pediatr Dermatol*. 2019;36(4):e95–e96. doi: 10.1111/pde.13825 31070265

[3] Ward KM, Barnett C, Fox LP, Grossman ME. Eyelash trichomegaly associated with systemic tacrolimus [8]. *Arch* Dermatol. 2006;142(2):248. doi: 10.1001/archderm.142.2.248 16490862

[4] Eisenberg DL, Toris CB, Camras CB. Bimatoprost and Travoprost: A Review of Recent Studies of Two New Glaucoma Drugs. Surv Ophthalmol. 2002 Aug 1;47(4 SUPPL. 1):S105–15. doi: 10.1016/s0039-6257(02)00327-2 12204706

[5] Tauchi M, Fuchs TA, Kellenberger AJ, Woodward DF, Paus R, Lütjen-Drecoll E. Characterization of an in vivo model for the study of eyelash biology and trichomegaly: Mouse eyelash morphology, development, growth cycle, and anagen prolongation by bimatoprost. *Br J Dermatol*. 2010;162(6):1186–1197. doi: 10.1111/j.1365-2133.2010.09685.x 20346040

[6] Maka V V., Rajanna H, Narasiyappah AK, Chitrapur R, Kilara N. Epidermal growth factor receptor inhibitors related trichomegaly of Eyelashes. *Oxford Med Case Reports*. 2014;2014(5):98–99. doi: 10.1093/omcr/omu038 25988043 PMC4360295

[7] Lane K, Goldstein SM. Erlotinib-associated trichomegaly. *Ophthal Plast Reconstr Surg*. 2007;23(1):65–66. doi: 10.1097/IOP.0b013e31802d9802 17237698

[8] Wang SB, Lei KJ, Liu JP, Jia YM. Eyelash trichomegaly following treatment with erlotinib in a non-small cell lung cancer patient: A case report and literature review. *Oncol Lett*. 2015;10(2):954–956. doi: 10.3892/ol.2015.3265 26622603 PMC4509027

[9] Pascual JC, Bañuls J, Belinchon I, Blanes M, Massuti B. Trichomegaly following treatment with gefitinib (ZD1839) [6]. *Br J Dermatol*. 2004;151(5):1111–1112. doi: 10.1111/j.1365-2133.2004.06265.x 15541102

[10] Goyal A, Blaes A. Trichomegaly Associated with Panitumumab. 2020;383(16):e94. doi: 10.1056/NEJMicm2003622 33053288

[11] Vaccaro M, Pollicino A, Barbuzza O, Guarneri B. Trichomegaly of the eyelashes following treatment with cetuximab. *Clin Exp Dermatol*. 2009;34(3):402–403. doi: 10.1111/j.1365-2230.2008.02842.x 19120397

[12] Borkar DS, Lacouture ME, Basti S. Spectrum of ocular toxicities from epidermal growth factor receptor inhibitors and their intermediate-term follow-up: A five-year review. *Support Care Cancer*. 2013;21(4):1167–1174. doi: 10.1007/s00520-012-1645-y 23151647

[13] Hutchison DM, Duffens A, Yale K, Park A, Cardenas K, Mesinkovska NA. Eyelash trichomegaly: a systematic review of acquired and congenital aetiologies of lengthened lashes. J Eur Acad Dermatol Venereol. 2022 Apr;36(4):536–546. doi: 10.1111/jdv.17877 34919300

[14] Johnstone MA. Hypertrichosis and increased pigmentation of eyelashes and adjacent hair in the region of the ipsilateral eyelids of patients treated with unilateral topical latanoprost. *Am J Ophthalmol*. 1997;124(4):544–547. doi: 10.1016/s0002-9394(14)70870-0 9323945

[15] Chen CS, Wells J, Craig JE. Topical prostaglandin f2α analog induced poliosis. *Am J Ophthalmol*. 2004;137(5):965–966. doi: 10.1016/J.AJO.2003.11.020 15126178

