# Expression of Concern: Assessing clinical and morphological features of megalotrichosis induced by Tyrosine kinase inhibitors versus Prostaglandins analogues

**DOI:** 10.1371/journal.pone.0320533

**Published:** 2025-03-07

**Authors:** The PLOS One Editors

The *PLOS One* Editors issue this Expression of Concern due to issues identified after publication of this article [1] about compliance with data-related requirements specified in the study’s ethics approval documentation.

Additionally, the article was republished on January 16, 2025, to address an error. Please download this article again to view the correct version.

The article’s Data Availability statement is updated to: All relevant data are within the paper and its Supporting Information files.

## Supporting information

S1 Table Clinical and morphological features of MT patients. (XLSX)
